# Coexistence of Fe^2+^ and Mn^2+^ inhibits nitrate removal in sulfur autotrophic denitrification systems

**DOI:** 10.3389/fmicb.2026.1739270

**Published:** 2026-02-25

**Authors:** Peng Ling Chen, Xue Jiao Huang, Zhao Jie Jiang, Xiao Fang Nong, Chun Min Xie

**Affiliations:** 1 Guangxi Key Laboratory of Agro-Environment and Agro-Products Safety, College of Agriculture, Guangxi University, Nanning, China; 2 Guangxi Bossco Environmental Technology, Nanning, China

**Keywords:** divalent iron, divalent manganese, inorganic electron donor, nitrate, sulfur autotrophic denitrification

## Abstract

Sulfur autotrophic denitrification (SAD) is commonly utilized for nitrate (NO_3_^−^-N) removal from groundwater because of its efficiency, minimal sludge production, cost-effectiveness, and carbon source independence. However, elevated Fe^2+^ and Mn^2+^ concentrations in groundwater may influence its efficiency. The purpose of this work was to explore the effects of coexisting Fe^2+^ and Mn^2+^ at varying 5 mM ratios on SAD efficiency and its underlying mechanisms. The results showed that adding 5 mM Fe^2+^ and Mn^2+^ at different ratios inhibited NO_3_^−^-N removal, reducing efficiency from 92.73% (without Fe^2+^/Mn^2+^) to 60.96% (Fe^2+^: Mn^2+^ = 9:1) by Day 6. All the systems with coexisting Fe^2+^ and Mn^2+^ accumulated NO_2_^−^-N and N_2_O. The generation of SO_4_^2−^ by the system gradually diminished, the Fe^2+^ removal rate gradually decreased, and the Mn^2+^ removal rate gradually increased as Fe^2+^ and Mn^2+^ concentrations increased and decreased, respectively. The coexistence of Fe^2+^ and Mn^2+^ reduced pH, decreased the relative abundance of *Thiobacillus*, and downregulated the expression of key denitrification (*nirS*, *norB*, *nosZ*) and sulfur oxidation (*dsrA*, *soxB*) genes, thereby compromising the denitrification efficiency of the SAD system. The rate-limiting reactions for system denitrogenation with Fe^2+^ and Mn^2+^ coexistence included NO reduction and N_2_O reduction. Furthermore, the key driving factors were the *nosZ*/*narG*, *nosZ*/*nirK*, *norB*/*nirK*, *dsrA*/*16S rRNA*, *soxB*/*nirK*, and *soxB*/*nirK* gene ratios. The findings of this study provide theoretical support for employing SAD technology to remove NO_3_^−^-N from water with elevated levels of coexisting Fe^2+^ and Mn^2+^.

## Graphical abstract

**Figure:**
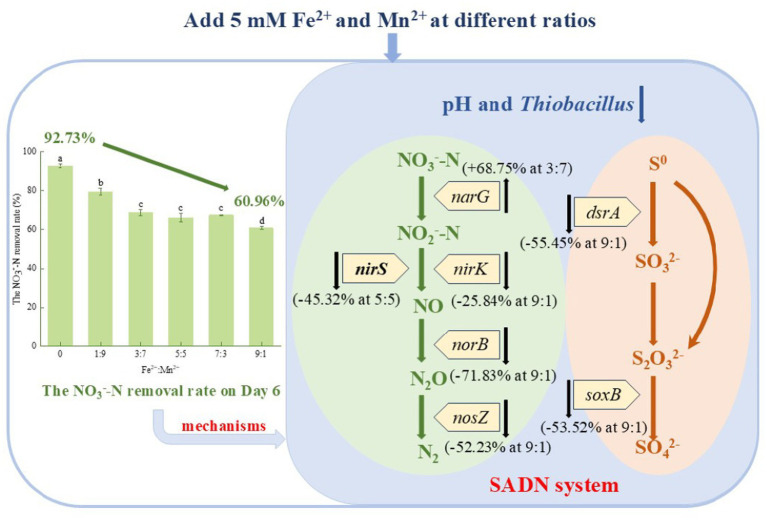

## Highlights

- Coexistence of 5 mM Fe^2+^ and Mn^2+^ inhibited the SAD system from removing NO_3_^−^-N.
- SAD system with coexisting Fe^2+^ and Mn^2+^ accumulated NO_2_^−^-N and N_2_O.
- SAD system’s key bacteria *Thiobacillus* and pH decrease with rising Fe^2+^ and falling Mn^2+^.
- Sulfur-oxidizing and denitrifying gene abundances decreased with coexisting Fe^2+^ and Mn^2+^.

## 1 Introduction

Nitrate (NO_3_^−^-N) is a primary cause of groundwater contamination. It is characterized by high solubility, rapid migration, and chemical stability in water bodies (Niu et al., 2021). Elevated NO_3_^−^-N concentrations contribute to environmental issues, such as algal blooms and water eutrophication, substantially affecting water quality and safety. Additionally, high NO_3_^−^-N concentrations in water bodies are associated with human health risks, including blue baby syndrome, methemoglobinemia, and esophageal cancer (Zhang and Wang, 2020). Given the urgent need to reduce total N emissions, effective NO_3_^−^-N removal remains a critical challenge in wastewater treatment (Onodera et al., 2021). Biological denitrification, an environmentally friendly, highly efficient, and economically viable approach, utilizes microorganisms to convert NO_3_^−^-N to N_2_ without generating nitrogenous wastes (D’Aquino et al., 2023). One of the bioremediation methods for removing nitrates, denitrification, is usually categorized into heterotrophic and autotrophic processes depending on the electron donor that is employed (Bai et al., 2020a; Wang et al., 2025).

Heterotrophic denitrification, widely applied because of its high N removal efficiency and rapid reaction rate, has considerable shortcomings, including additional carbon source requirements, excessive sludge production, and high CO_2_ emissions (Han et al., 2025; Xu et al., 2021). These limitations pose challenges in meeting carbon emission reduction targets and carbon neutrality goals. Conversely, autotrophic denitrification, particularly sulfur autotrophic denitrification (SAD), offers specific advantages, such as minimal sludge yield, low CO_2_ emissions, and external carbon source independence (Wang et al., 2025; D’Aquino et al., 2023; Bai et al., 2020a). SAD has been frequently employed to remove NO_3_^−^-N from groundwater and municipal tailwater (Wang T. et al., 2023). Because the elemental sulfur (S^0^) has the advantage of low cost, ease of handling and transportation, and high denitrification efficiency, it has been extensively utilized as an electron donor in SAD (Zhao et al., 2024). The stoichiometric formula for denitrification with S^0^ as the electron donor is shown below (Equation 1) (Zhao et al., 2024):


$$\begin{array}{l} S^{0}+0.87{{\mathrm{6NO}}_{3}}^{-}+0.34{\mathrm{3CO}}_{2}+0.37{{\mathrm{9HCO}}_{3}}^{-}+0.02{\mathrm{3CO}}_{2}\\ +0.08{{\mathrm{NH}}_{4}}^{+}\to 0.08C_{5}H_{7}O_{2}N+0.82{\mathrm{4H}}^{+}+0.44N_{2}+{{\mathrm{SO}}_{4}}^{2-} \end{array}$$ (1)

Notably, excess NO_3_^−^-N is frequently accompanied by high Fe^2+^ and Mn^2+^ concentrations in natural groundwater bodies (Genuchten and Ahmad, 2020). Fe^2+^ plays crucial roles in bacterial growth, microbial oxygen transfer, electron transport, and functional protein synthesis. However, excessive Fe^2+^ can exert toxic and inhibitory effects on microorganisms (Jiang et al., 2023). Wen et al. (2019) found that while low Fe^2+^ concentrations facilitated the removal of total dissolved solids (TDS) and NO_3_^−^-N, high Fe^2+^ concentrations inhibited their removal. Similarly, Bai et al. (2020a) evaluated the denitrification performance of strain HY129 in wastewater containing NO_3_^−^-N and Mn^2+^ and observed that although high Mn^2+^ concentrations inhibited the reduction of NO_2_^−^-N and caused nitrite accumulation, low Mn^2+^ concentrations could promote microbial denitrification. In addition to its microbial effects, excessive Mn^2+^ exposure poses human health risks, including coronary heart disease (Bai et al., 2020b), delayed reproductive maturity, and neurological disorders in children (Khan et al., 2012). Groundwater Mn^2+^ concentrations can reach up to 2,300 μg/L in certain regions (Ying et al., 2017).

The preliminary investigations indicated that both 5 mM Fe^2+^ and 5 mM Mn^2+^ impeded the denitrification process in the SAD system. Although Fe^2+^ and Mn^2+^ are frequently detected in wastewater with high NO_3_^−^-N concentrations, the impact of their coexistence on denitrification in SAD systems is currently unknown. In this study, we investigated the effects of 5 mM Fe^2+^ and Mn^2+^ coexisting in varying ratios on the denitrification ability of SAD in terms of S and N products by conducting batch experiments with S^0^ as the electron donor. Additionally, we analyzed changes in microbial community structure and relative functional gene expression to examine the response patterns between microbial community structure, related functional gene expression, and S and N products, thereby clarifying the potential mechanisms. The findings of this study can theoretically facilitate NO_3_^−^-N removal from water containing Fe^2+^ and Mn^2+^ via SAD technology.

## 2 Materials and methods

### 2.1 Bacterial enrichment and culture

The Xingchang Wastewater Treatment Plant in Zhejiang, China, provided the sludge that was used to enrich and cultivate SAD-associated microorganisms. The sludge was cultivated with an enrichment culture solution comprising 0.62 g/L Na_2_S_2_O_3_·5H_2_O, 0.36 g/L KNO_3_, 0.21 g/L NaHCO_3_, 0.34 g/L KH_2_PO_4_, 0.086 g/L NH_4_Cl, 0.076 g/L MgCl_2_, 0.003 g/L CaCl_2_, and 0.25 mL/L trace elements (Pang and Wang, 2021). The trace element compositions included 0.1 mg/L FeSO_4_·7H_2_O, 0.03 mg/L H_3_BO_3_, 0.12 mg/L MnCl_2_·4H_2_O, 0.12 mg/L CoCl_2_·6H_2_O, 0.024 mg/L NiCl_2_·6H_2_O, 0.07 mg/L ZnCl_2_, 0.015 mg/L CuSO_4_·5H_2_O, and 0.036 mg/L Na_2_MoO_4_·2H_2_O. Serum bottles (500 mL), each containing 250 mL of the enriched culture fluid and 100 mL of sludge, were used to domesticate the sludge. Following N_2_ flushing for 15 min, the bottles were promptly sealed to establish an anaerobic environment. According to previous studies, S^0^ oxidation under anaerobic conditions can proceed through the following pathways (Pang and Wang, 2021): (1) Biological conversion of S^0^ to SO_3_^2−^ (Equation 2); (2) Reaction of S^0^ with SO_3_^2−^ to form S_2_O_3_^2−^(Equation 3); and (3) Biological oxidation of S_2_O_3_^2−^ to SO_4_^2−^ (Equation 4).


$$S^{0}+{\mathrm{3H}}_{2}O\to {{\mathrm{SO}}_{3}}^{2-}+{\mathrm{4e}}^{-}+{\mathrm{6H}}^{+}$$ (2)

$$S^{0}+{{\mathrm{SO}}_{3}}^{2-}\to S_{2}{O_{3}}^{2-}$$ (3)

$$S_{2}{O_{3}}^{2-}+{\mathrm{5H}}_{2}O\to {{\mathrm{2SO}}_{4}}^{2-}+{\mathrm{8e}}^{-}+10H^{+}$$ (4)

### 2.2 Experimental design

Artificial water distribution was employed in the experiments to simulate natural groundwater, comprising 0.45 g/L KNO_3_, 0.14 g/L KH_2_PO_4_, 0.03 g/L MgCl_2_, 0.001 g/L CaCl_2_, 0.42 g/L NaHCO_3_, and 0.1 mL/L trace elements (Pang and Wang, 2021). Each of the 100 mL serum vials was filled with 50 mL of artificially made water. Moreover, 2.5 mM Fe^2+^, 2.5 mM Mn^2+^, and 0.05 g S^0^ were added to the blank control group (CK) to assess the interactions of Mn^2+^, Fe^2+^, and S^0^ in the absence of microorganisms. Each experimental group received 0.05 g of S^0^ and 2 g of cultivated anaerobic sludge following centrifugation of the sludge for 20 min at 5,000 rpm. Five experimental groups and a control group which did not contain Fe^2+^ or Mn^2+^ additions were established (Zeng et al., 2024). The initial Fe^2+^: Mn^2+^ ratios in the five experimental groups were as follows: 1:9 (0.5 mM:4.5 mM), 3:7 (1.5 mM:3.5 mM), 5:5 (2.5 mM:2.5), 7:3 (3.5 mM:1.5 mM), 9:1 (4.5 mM:0.5 mM), 7:3 (3.5 mM:1.5 mM), and 9:1 (4.5 mM:0.5 mM). All experimental and control groups were conducted in triplicate (Zeng et al., 2024). To create an anaerobic environment, each serum vial was sealed with a cap after 15 min of N_2_ washing. The mixture was subsequently incubated in a thermostatic shaking chamber for 12 d at 30 °C and 150 rpm. The NO_3_^−^-N, NO_2_^−^-N, NH_4_^+^-N, N_2_O, Fe^2+^, Mn^2+^, and SO_4_^2−^ concentrations were assessed by sampling the supernatants on Days 1, 2, 4, 6, 8, 10, and 12. On Day 12, samples underwent *16S rRNA* high-throughput sequencing and functional gene abundance assays, while the pH of the supernatant was assessed on Days 1 and 12.

### 2.3 Indicators and methods of analysis

#### 2.3.1 Measurement of water quality indicators

A 0.22 μm filter membrane was used to filter water samples before water quality indicators were measured. UV spectrophotometry, N-(1-naphthalene)-ethylenediamine spectrophotometry, indophenol blue colorimetry, and barium chromate spectrophotometry were used to quantify the NO_3_^−^-N, NO_2_^−^-N, NH_4_^+^-N, and SO_4_^2−^ concentrations in the water samples, respectively (Huang et al., 2024). The N_2_O concentration was measured using a gas chromatograph (7890A, Agilent, CA, United States) (Pang and Wang, 2021). An inductively coupled plasma emission spectrometer (ICP-5000, Beijing Spotlight Science and Technology Co., Ltd.) was used to measure the concentrations of Fe^2+^ and Mn^2+^ in the water samples (Zeng et al., 2024). A pH meter (S220, Mettler Toledo Co., Ltd., OH, United States) was employed to measure the value of pH in the water samples.

#### 2.3.2 High-throughput sequencing analysis

Following a 12-d incubation period, samples were collected, and genomic DNA was extracted using the DNA kit (E.Z.N.a.^®^ Soil DNA Kit, Omega Bio-tek, Norcross, GA, United States). the NanoDrop quantification technique was employed to test the DNA concentration and purity, and the samples were subsequently delivered to Shanghai Parsonage Bioscience Co. Ltd. for Illumina MiSeq sequencing. PCR was performed using primers 338F (5′ -ACTCCTACGGGAGGCAGCA-3′) and 806R (5′ -GGACTACHVGGGTWTCTAAT-3′) to amplify the V3–V4 region of the bacterial *16S rRNA* gene (Pang and Wang, 2021). Following PCR products high-throughput sequencing, the identified sequences were clustered into operational taxonomic units (OTUs) after shearing and optimization with 97% sequence identity. OTU clustering was employed to statistically analyze community composition and structure at the genus and phylum levels.

#### 2.3.3 Functional gene abundance assay

Utilizing *rpoB* as an internal reference gene, quantitative real-time polymerase chain reaction (qPCR) was employed to assess the abundance of functional genes associated with sulfur oxidation (*dsrA* and *soxB*) and denitrification (*narG*, *nirK*, *nirS*, *norB*, and *nosZ*). The 10 μL qPCR system comprised 5 μL of TB Green premix Ex Taq II (Tli RNaseH Plus) (2X), 1 μL of DNA, 3.2 μL of sterile water, 0.4 μL of forward primer, and 0.4 μL of reverse primer (Bai et al., 2020a). The forward and reverse primers for the corresponding genes are listed in Supplementary Table S1.

#### 2.3.4 Processing and analyzing data

To process the experimental data, Microsoft Excel 2016 was utilized, Origin 2018 (OriginLab Corp., Northampton, MA, United States) was used to make the figures. Used SPSS 22.0 (IBM Corp., Armonk, NY, United States) to perform one-way analysis of variance (ANOVA) and follow Duncan’s Multiple Range Test, with *p* < 0.05 regarded as statistically significant.

## 3 Results and discussion

### 3.1 Effects of Fe^2+^ and Mn^2+^ coexistence on nitrogen transformation in the SAD system

**Figure 1:**
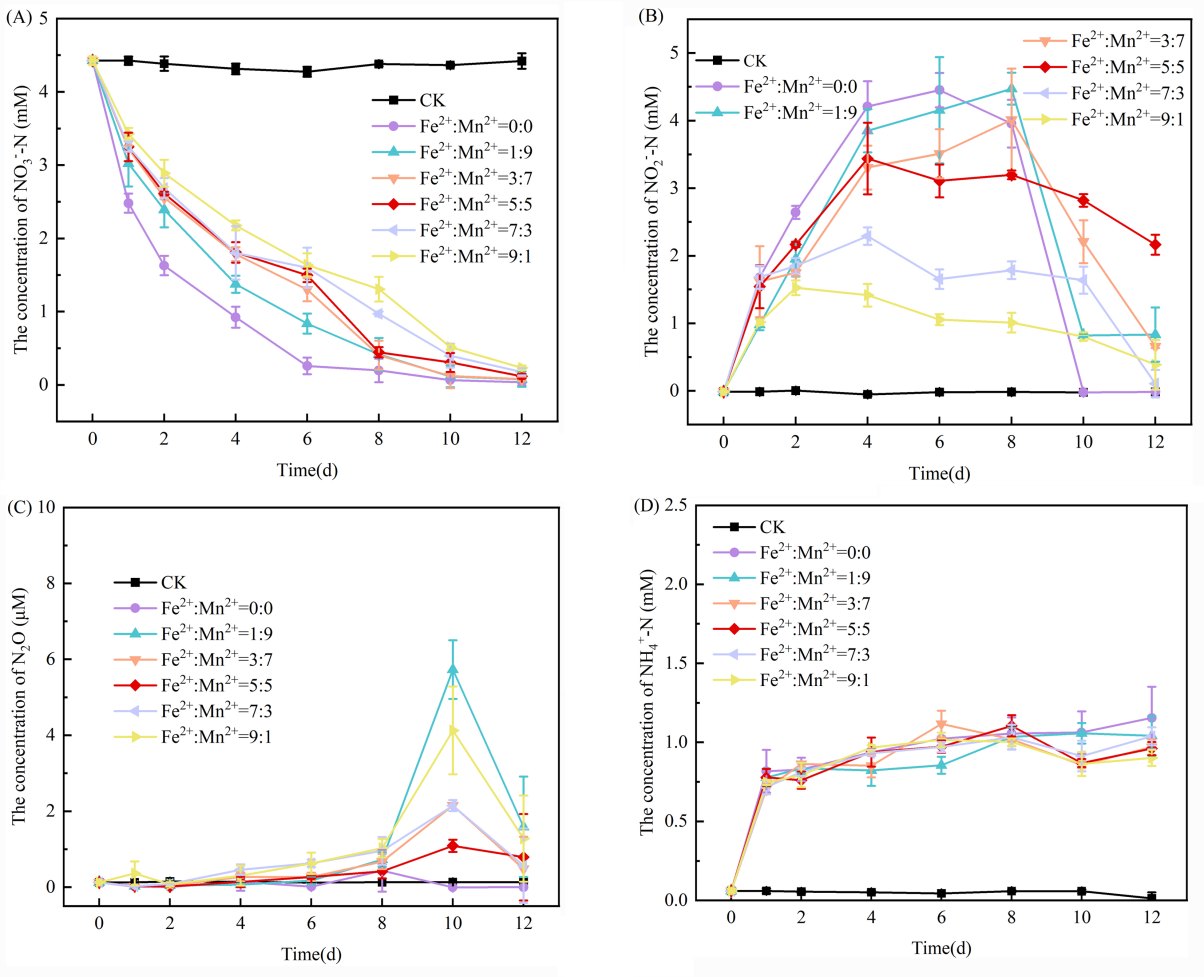
Changes in the concentrations of NO_3_^−^-N **(A)**, NO_2_^−^-N **(B)**, N_2_O **(C)**, and NH_4_^+^-N **(D)** in the SAD system with coexisting Fe^2+^ and Mn^2+^.

Figure 1A demonstrates how the coexistence of various Fe^2+^ and Mn^2+^ ratios affects the NO_3_^−^-N concentration in the SAD system. The NO_3_^−^-N concentration in systems with varying 5 mM Fe^2+^ and Mn^2+^ ratios were higher than that in the system without Fe^2+^ and Mn^2+^, indicating that the coexistence of 5 mM Fe^2+^ and Mn^2+^ inhibited the removal of NO_3_^−^-N in the SAD system. As the Fe^2+^ concentration increased, the NO_3_^−^-N removal of the SAD system steadily declined. On the sixth day of incubation, the NO_3_^−^-N removal rate from the system decreased significantly from 92.73% without adding Fe^2+^ and Mn^2+^ to 60.96% when an Fe^2+^: Mn^2+^ ratio of 9:1 was applied. Zeng et al. (2024) found that an SAD system with an Fe^2+^: Mn^2+^ ratio of 0:20 achieved a 90.91% NO_3_^−^-N removal rate. Similarly, the removal rate of NO_3_^−^-N decreased substantially to 86.15% when the Fe^2+^: Mn^2+^ ratio reached 5:5, indicating that NO_3_^−^-N removal declined with increasing Fe^2+^ concentration. High Fe^2+^ concentrations may result in this behavior by lowing the pH of the system and adversely affecting microorganisms (Wang et al., 2020). This reduces the effectiveness of the SAD system in removing NO_3_^−^-N by limiting the growth of associated denitrifying microbes. Nonetheless, by the 12th day of incubation, the NO_3_^−^-N removal rate in all SAD systems with varying Fe^2+^ and Mn^2+^ ratios exceeded 94%. This phenomenon can be attributed to the declines in both Fe^2+^ and Mn^2+^ concentrations toward the conclusion of the reaction, thereby reducing toxicity to denitrifying microorganisms. Additionally, microbial adaptation to the environment with coexisting Fe^2+^ and Mn^2+^ facilitated the effective removal of NO_3_^−^-N from the system.

Both NO_2_^−^-N and N_2_O serve as typical intermediate products of denitrification. N_2_O functions as a greenhouse gas with potentially detrimental effects on the ozone layer, whereas low levels of NO_2_^−^-N accumulation can also limit microbial activity (Albina et al., 2019). In the pre-experimental phase, the NO_2_^−^-N concentrations in systems with varying Fe^2+^ and Mn^2+^ ratios were lower than those in the systems without any additions. Additionally, these concentrations decreased as Fe^2+^ concentration increased (Figure 1B). Fe^2+^ is more prone to electron loss during redox processes, facilitating the NO_2_^−^-N reduction (Tekerlekopoulou et al., 2013). At the conclusion of the experiment, the system without Fe^2+^ and Mn^2+^ addition exhibited no NO_2_^−^-N accumulation, whereas the systems with 5 mM Fe^2+^ and Mn^2+^ addition at various ratios displayed various levels of NO_2_^−^-N accumulation. The system with an Fe^2+^: Mn^2+^ ratio of 5:5 exhibited the highest NO_2_^−^-N accumulation at 2.16 mM. The NO_2_^−^-N and N_2_O concentrations of all experimental systems showed an initially increasing and subsequently decreasing trend (Figures 1B,C). This can be explained by the high NO_3_^−^-N concentration in the initial phases of the experiment, which prevents denitrification and causes temporary NO_2_^−^-N and N_2_O accumulation (Li et al., 2023). Luo et al. (2018) indicated that the NO_2_^−^-N reduction rate increased only when *Pseudomonas* sp. H117 cells were almost completely exhausted with 10 mg/L NO_3_^−^-N. This suggests that high NO_3_^−^-N concentrations inhibit the NO_2_^−^-N reduction process. At the conclusion of the experiment, no N_2_O accumulation occurred in the system without Fe^2+^ and Mn^2+^ addition, whereas the systems with Fe^2+^ and Mn^2+^ addition had different degrees of N_2_O accumulation. Among these, the system with an Fe^2+^: Mn^2+^ ratio of 1:9 had the highest N_2_O accumulation at 1.59 μM. In conclusion, insufficient denitrification in the SAD system may result from introducing 5 mM varying Fe^2+^ and Mn^2+^ ratios, potentially leading to NO_2_^−^-N and N_2_O accumulation (Wang et al., 2022).

In a system containing 5 mM of coexisting Fe^2+^ and Mn^2+^ in varying ratios, the NH_4_^+^-N concentration in the system initially increased and subsequently decreased, exhibiting the highest rate of increase on the first day and then leveling off (Figure 1D). Protein hydrolysis in sludge can yield NH_4_^+^-N, with the sharp increase in NH_4_^+^-N during the 1st phase attributed to the increase in total protein and elevated hydrolysis levels observed during the early period of increased sludge concentration (Jiang et al., 2024). The decrease in NH_4_^+^-N levels in the later stages may be attributed to the sludge entering the methanogenic stage, characterized by a reduction in protein content drops and partial consumption of NH_4_^+^-N by microbial growth. The system without Fe^2+^ and Mn^2+^ addition generated the most NH_4_^+^-N, potentially because the addition of 5 mM varying Fe^2+^ and Mn^2+^ ratios inhibited sludge hydrolysis. Fe^2+^ is essential for the heme c synthesis, which aids anammox metabolism by facilitating the production of associated enzymes, including hydroxylamine oxidoreductase (HAO), nitrite reductase (NIR), hydrazine synthase (HZS), and hydrazine dehydrogenase (Kartal and Keltjens, 2016). In the system with varying Fe^2+^ and Mn^2+^ ratios, NH_4_^+^-N production initially decreased and subsequently increased as the Fe^2+^ concentration increased. This is likely because a low Fe^2+^ concentration increases the related enzyme activity, thereby promoting anammox activity and increasing NH_4_^+^-N utilization. Conversely, a high Fe^2+^ concentration inhibits anammox activity. Jiang et al. (2023) found that Fe^2+^ toxicity and the inhibition of anammox activity occur at Fe^2+^ concentrations of 70–80 mg/L in anammox bacteria. Sindhu et al. (2021) also demonstrated that 5 mM Fe^2+^ was detrimental to anammox bacteria, whereas 1 mM Fe^2+^ enhanced anammox activity and increased anammox bacteria quantities.

### 3.2 Effects of Fe^2+^ and Mn^2+^ coexistence on SO_4_^2−^ concentration and pH in the SAD system

**Figure 2:**
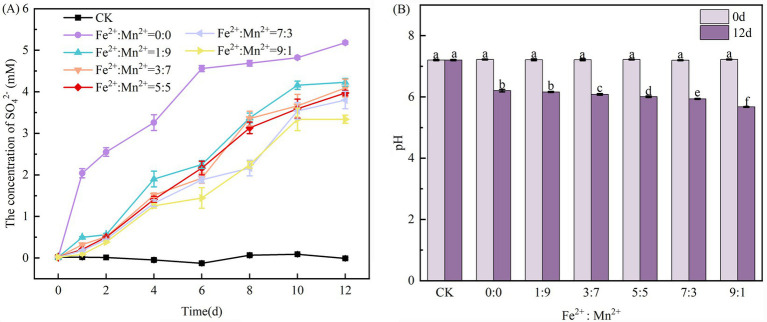
Changes in the concentration of SO_4_^2−^ **(A)** and pH **(B)** in the SAD system with coexisting Fe^2+^ and Mn^2+^.

The SO_4_^2−^ concentration increased as the NO_3_^−^-N concentration decreased, suggesting that sulfur oxidation and NO_3_^−^-N reduction was coupled (Pang and Wang, 2021). Theoretically, 7.54 mg of SO_4_^2−^ is produced for every 1 mg of NO_3_^−^-N eliminated (Zhao et al., 2024). In this research, the maximum theoretical SO_4_^2−^ production was observed in the system without Fe^2+^ and Mn^2+^ addition (Figure 2A). The yield of SO_4_^2−^ and removal rate of NO_3_^−^-N were positively correlated; the higher the removal rate of NO_3_^−^-N, the more SO_4_^2−^ was generated (Wang et al., 2019). When 5 mM Fe^2+^ and Mn^2+^ were added to the system, SO_4_^2−^ production decreased as the Fe^2+^ concentration increased, with the levels far below those predicted. In addition, Figure 1A shows that NO_3_^−^-N removal declined as the Fe^2+^ concentration increased in all treatments. Accompanying this decline, the production of SO_4_^2−^ also decreased. This suggests that the SAD system for all treatments exhibits NO_3_^−^-N reduction coupled with sulfur oxidation, which aligns with the results of earlier research (Wang et al., 2019). Although SO_4_^2−^ is not inherently toxic, excessive consumption can lead to organ damage in humans, and high SO_4_^2−^ emissions may considerably disrupt ecosystem stability.

pH can influence the denitrification rate by altering the charged state of substrates and bacterial enzyme proteins within the culture system, thereby influencing nutrient absorption by cells and the corresponding enzyme activity (Song T. et al., 2021). The ideal pH range for denitrification is 6–8. Beyond this range, the N removal rate may be limited, leading to potential NO_2_^−^-N and N_2_O accumulation (Baeseman et al., 2006). In the absence of Fe^2+^ and Mn^2+^ addition, the pH of the SAD system significantly decreased at the conclusion of the incubation process. When various Fe^2+^ and Mn^2+^ ratios were added, the pH of the system decreased more considerably as the Fe^2+^ concentration increased (Figure 2B), with a minimum value of 5.67 observed at Fe^2+^: Mn^2+^ = 9:1. The above phenomenon could be because Fe^2+^ is a propensity to lose electrons during a redox process, resulting in the formation of H^+^. Additionally, introducing a small quantity of oxygen during sampling can lead to the Fe^2+^ oxidating to Fe^3+^ and subsequent hydrolysis, resulting in a lower pH.

In the absence of microbes, Mn^2+^, Fe^2+^, and S^0^ showed no observable reaction (Figure 2A). Upon the addition of Fe^2+^, several biochemical reactions may occur (Pang and Wang, 2021; Chen et al., 2024): (1) Fe^2+^ can react with NO_3_^−^-N if iron-autotrophic denitrifying bacteria become enriched (Equation 5); (2) Fe^2+^ is oxidized to Fe^3+^ by oxygen (Equation 6); (3) The resulting Fe^3+^ may react with S^0^ (Equation 7); and (4) Fe^3+^ can hydrolyze to form Fe(OH)_3_ (Equation 8). Similarly, the addition of Mn^2+^ may lead to the following reactions (Bai et al., 2022; Yu and Leadbetter, 2020): (1) Mn^2+^ can react with NO_3_^−^-N upon later enrichment of manganese-autotrophic denitrifying bacteria (Equation 9); (2) The generated MnO_2_ may react with S_2_O_3_^2−^ (Equation 10); and (4) In an anoxic manganese-rich environment, MnO_2_ can also react with NH_4_^+^ (Equations 11, 12).


$$10{\mathrm{Fe}}^{2+}+{{\mathrm{2NO}}_{3}}^{-}+24H_{2}O\to 10\mathrm{Fe}{\left(\mathrm{OH}\right)}_{3}+N_{2}+18H^{+}$$ (5)

$${\mathrm{4Fe}}^{2+}+O_{2}+{\mathrm{4H}}^{+}\to {\mathrm{4Fe}}^{3+}+{\mathrm{2H}}_{2}O$$ (6)

$${\mathrm{6Fe}}^{3+}+S^{0}+{\mathrm{4H}}_{2}O\to {\mathrm{6Fe}}^{2+}+{{\mathrm{SO}}_{4}}^{2-}+{\mathrm{8H}}^{+}$$ (7)

$${\mathrm{Fe}}^{3+}+{\mathrm{3H}}_{2}O\to \mathrm{Fe}{\left(\mathrm{OH}\right)}_{3}+{\mathrm{3H}}^{+}$$ (8)

$${\mathrm{5Mn}}^{2+}+{{\mathrm{2NO}}_{3}}^{-}+{\mathrm{4H}}_{2}O\to {\mathrm{5MnO}}_{2}+N_{2}+{\mathrm{8H}}^{+}$$ (9)

$${\mathrm{4MnO}}_{2}+S_{2}{O_{3}}^{2-}+{\mathrm{6H}}^{+}\to {\mathrm{4Mn}}^{2+}+{{\mathrm{2SO}}_{4}}^{2-}+{\mathrm{3H}}_{2}O$$ (10)

$${\mathrm{3MnO}}_{2}+{{\mathrm{2NH}}_{4}}^{+}+{\mathrm{4H}}^{+}\to {\mathrm{3Mn}}^{2+}+N_{2}+{\mathrm{6H}}_{2}O$$ (11)

$${\mathrm{4MnO}}_{2}+{{\mathrm{NH}}_{4}}^{+}+{\mathrm{6H}}^{+}\to {\mathrm{4Mn}}^{2+}+{{\mathrm{NO}}_{3}}^{-}+{\mathrm{5H}}_{2}O$$ (12)

### 3.3 Variations in Fe^2+^ and Mn^2+^ in the SAD systems

**Figure 3:**
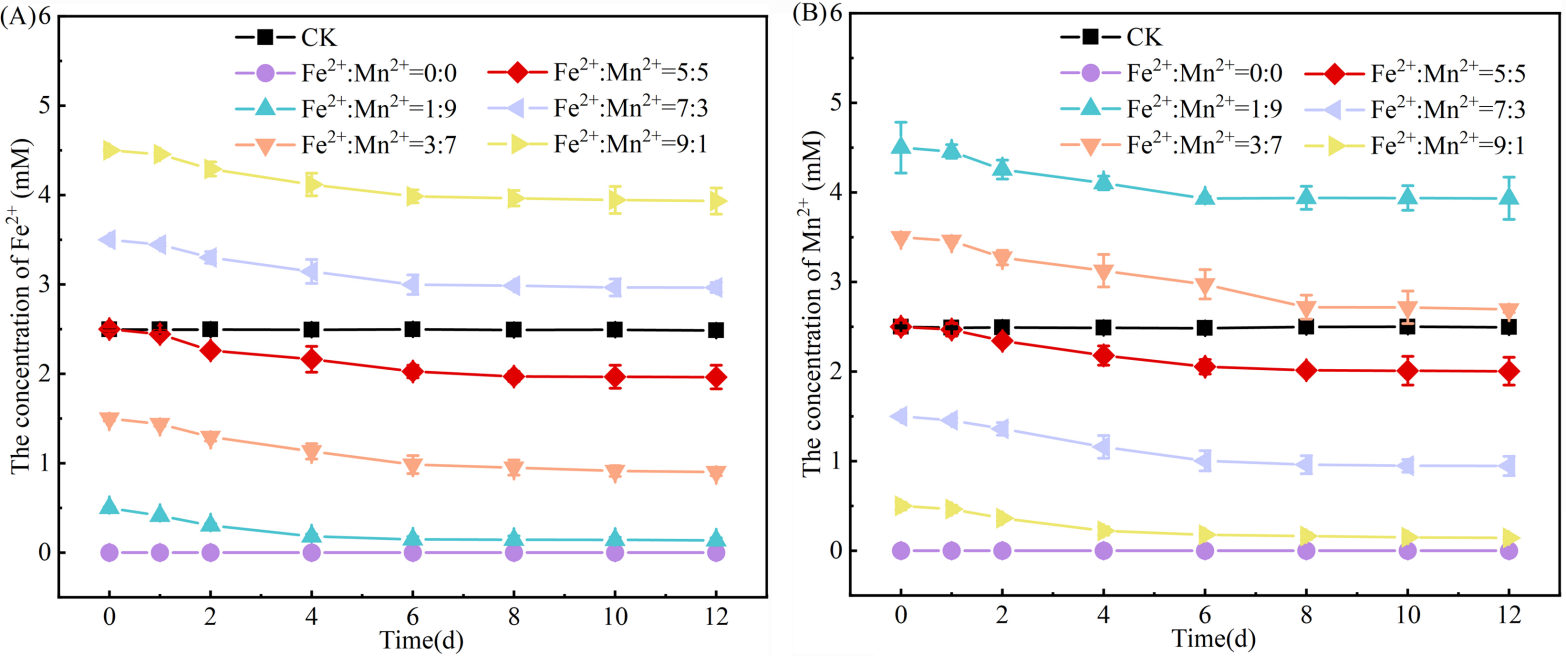
Changes in the concentrations of Fe^2+^ **(A)** and Mn^2+^ **(B)** in the system with coexisting Fe^2+^ and Mn^2+^.

Both Fe^2+^and Mn^2+^ can act as electron donors for denitrification because they release electrons during oxidative processes (Wang Y. N. et al., 2023). Upon completion of the experiment, the concentrations of Fe^2+^ and Mn^2+^ decreased in all systems, regardless of the initial Fe^2+^/Mn^2+^ ration. As the proportion of Fe^2+^ increased and that of Mn^2+^ decreased, the removal rate of Fe^2+^ progressively declined, while that of Mn^2+^ gradually rose (Figures 3A,B). Bai et al. (2020a) discovered that increasing the initial concentration of Mn^2+^ from 5 to 60 mg/L substantially decreased Mn^2+^ removal from 95.79 to 75.53% via the denitrifying strain *Cupriavidus* sp. HY129. The strain may exhibit increased activity, utilizing most of the Mn^2+^ ions for denitrification at low Mn^2+^ concentrations. However, high Mn^2+^ concentrations restrict bacterial activity, lower the rate of Mn^2+^ oxidation, and decrease electron release. Zhang et al. (2019) observed that excessive Mn^2+^ disrupted the equilibrium of the system that regulates the metabolism of reactive oxygen species, thereby decreasing biomass. Similarly, as the Fe^2+^ concentration increased, the microorganism activity was suppressed, reducing the removal of Fe^2+^ in the SAD system. According to Dixon et al. (2012), introducing large amounts of Fe^2+^ generates reactive oxygen species that damage cell and organelle membranes and inhibit microbial activity. Chang et al. (2021) reported that a low pH affects specific microbial functions and reduces Fe-oxidizing microorganism activity, thereby decreasing Fe^2+^ removal from the system. This corresponds to the significant decrease in pH along with the increase in the Fe^2+^ concentration.

In systems where S^0^, Fe^2+^ and Mn^2+^ coexist, all three can act as electron donors to drive denitrification. Denitrifying bacteria preferentially utilize electron donors with stronger reducing capacities. Among them, Fe^2+^ possesses the highest reducing power, with an electron release rate significantly exceeding that of S^0^, whereas Mn^2+^ exhibits the weakest reducing capacity, even lower than that of S^0^ (Lin et al., 2022). When S^0^, Fe^2+^ and Mn^2+^ are present together, denitrifying bacteria primarily activate the Fe^2+^ oxidation pathway to transfer electrons to NO_3_^−^. This suppresses S^0^ oxidation and limits the electron supply for sulfur autotrophic denitrification, ultimately lowering the nitrate reduction rate. Additionally, electrons released from Fe^2+^ oxidation can elevate the system’s oxidation–reduction potential (ORP), creating conditions unfavorable for the growth of sulfur autotrophic denitrifying bacteria (Yu et al., 2022). Such ORP shifts may also indirectly inhibit the activity of key denitrifying enzymes, such as nitrate reductase (nar) and nitrite reductase (nir), thereby impeding critical steps in NO_3_^−^-N reduction. Consequently, even in the presence of S^0^, the electron transport chain cannot function efficiently. Moreover, Fe^2+^ can rapidly transfer electrons to NO_3_^−^ by via direct binding to cytochrome *c* in the bacterial electron transport chain (Cui et al., 2021). In contrast, S^0^-mediated electron transfer requires sulfur oxidases (e.g., sulfide quinone oxidoreductase), which exhibit lower affinity for electron carriers compared to Fe^2+^ (Bobadilla Fazzini et al., 2013). Furthermore, Fe^3+^ produced from Fe^2+^ oxidation may react with S^0^ to form FeS precipitates that coat S^0^ particles, physically blocking contact between S^0^ and sulfur oxidases and further inhibiting electron release from S^0^. When these precipitates deposits on microbial surfaces, they can obstruct NO_3_^−^-N entry into cells and potentially hinder nutrient uptake and metabolism (Coby and Picardal, 2005). Therefore, in a coexisting system of Fe^2+^ and Mn^2+^, the NO_3_^−^-N removal rate of the SAD system progressively decreased with increasing Fe^2+^ concentration. In comparison, Mn^2+^ has a relatively minor influence on NO_3_^−^-N reduction, largely due to its lower reducing capacity.

### 3.4 Effects of Fe^2+^ and Mn^2+^ coexistence on microbial community structure in the SAD systems

**Figure 4:**
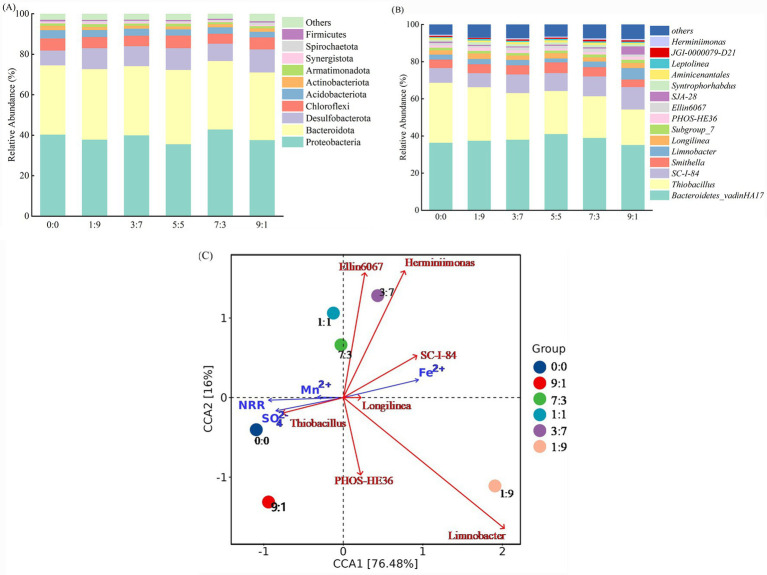
Relative abundance of bacterial communities at the phyla level **(A)**, genus level **(B)**, and correlation analysis (CCA) of genus-level denitrifying microorganisms **(C)** in the SAD system with coexisting Fe^2+^ and Mn^2+^.

In the SAD system without Fe^2+^ and Mn^2+^ addition, Proteobacteria (40.33%), Bacteroidetes (34.22%), Desulfobacterota (7.3%), Chloroflexi (6.03%), and Acidobacterota (4.03%) were the most predominant phyla (Figure 4A). Figure 4A shows that the system with varying Fe^2+^ and Mn^2+^ proportions was similar to the dominating phylum of the system absent Fe^2+^ and Mn^2+^. The most prevalent functional bacterial phylum of the SAD system was identified to be Proteobacteria, which are involved in the denitrification process (Lin et al., 2022; Liu et al., 2021). Proteobacteria remained the dominating phylum in the SAD system, exhibiting abundances of 37.83% (Fe^2+^: Mn^2+^ = 1:9), 39.96% (3:7), 35.58% (5:5), 42.85% (7:3), and 37.62% (9:1), despite a decline in their abundance with the addition of various Fe^2+^: Mn^2+^ ratios. Fe^2+^ and Mn^2+^ addition decreased NO_3_^−^-N removal from the SAD system, suggesting a positive correlation between the relative abundance of Proteobacteria and the decrease in the N removal rate. Desulfobacterota contributes approximately 12% of the N pathway genes, indicating its importance in the N cycle (Nie et al., 2021). Compared to the system without Fe^2+^ and Mn^2+^ addition, the addition of 5 mM of various ratios of both Fe^2+^ and Mn^2+^ increased the relative abundance of Desulfobacterota. These findings indicate that the coexisting Fe^2+^ and Mn^2+^ may encourage some aspects of N cycling and enhance the denitrification capacity of the system at the conclusion of the experiment. Chloroflexi can decompose soluble organic materials and chemicals that contribute to cellular degradation. They can also collaborate to form network architectures that promote the production of anaerobic ammonia oxidation particles (Kindaichi et al., 2012). Jiang et al. (2023) demonstrated that Fe^2+^ addition enhanced the settling of anammox sludge, promoted its granulation, and increased the quantity of Chloroflexi. However, in this study, the Chloroflexi abundance was lower in systems with different Fe^2+^ and Mn^2+^ ratios than in those without Fe^2+^ and Mn^2+^ addition. This could be explained by the addition of varying 5 mM Fe^2+^ and Mn^2+^ ratios, inhibiting the activity of anammox bacteria. Acidobacterota contributes to the Fe cycle by promoting the transformation of Fe^2+^ into Fe^3+^ (Lu et al., 2010). The relative abundance of Acidobacterota in systems with varying Fe^2+^ and Mn^2+^ ratios was lower than that in systems without Fe^2+^ and Mn^2+^ addition. This can be attributed to the coexistence of Fe^2+^ and Mn^2+^ inhibiting the oxidation of Fe^2+^ in the system.

The top 15 dominant genera at the genus level, including *Bacteroidetes-vadinHA17*, *Thiobacillus*, *SC-I-84*, *Limnobacter*, *Longilinea*, *PHOS-HE36*, *Ellin6067*, and *Herminiimonas*, were all associated with the N cycle, indicating that most genus-level microbial communities participated in denitrification and hydrolysis (Figure 4B). *Bacteroidetes_vadinHA17* is an unclassified anaerobic genus within the phylum Bacteroidetes. It can degrade complex organic matter in wastewater into readily available carbon sources, which can be utilized by denitrifying bacteria, thus indirectly improving nitrogen removal efficiency in wastewater bioremediation systems (Li et al., 2022; Zhang et al., 2022). The genus *Thiobacillus*, associated with sulfur-oxidizing bacteria (SOB), was found in all treatments. Previous studies have indicated that *Thiobacillus* ranks among the most prevalent SAD taxa identified in denitrifying systems across diverse settings (Xu et al., 2020). The relative abundance of *Thiobacillus* in the system without Fe^2+^ and Mn^2+^ reached 32.32% (Figure 4B). Yang et al. (2018) employed anaerobic sludge from a municipal wastewater treatment facility and thiosulfate as a substrate for enrichment culture studies. Their findings indicated that *Thiobacillus* was the dominant genus in sludge, with an autotrophic denitrification rate reaching 21 mg N_2_/(g VSS·d). In the system with coexisting 5 mM Fe^2+^ and Mn^2+^, the relative abundance of *Thiobacillus* gradually decreased as the Fe^2+^ concentration increased. Additionally, the SAD effect gradually diminished. The system with the lowest relative abundance of *Thiobacillus*, which had an Fe^2+^: Mn^2+^ ratio of 9:1, showed a decrease of 19.10%. Pang and Wang (2021) discovered that raising the Fe^2+^ concentration from 0 to 5 mM resulted in a dramatic reduction in the relative abundance of *Thiobacillus*, from 81.6 to 27.4%. The relative abundance of *SC-I-84*, an anammox bacterium (Song Y. P. et al., 2021), increased across all treatments as the Fe^2+^ concentration rose, indicating that Fe^2+^ addition enhanced anammox activity. Both *PHOS-HE36* and *Limnobacter* are denitrifying bacteria (Liang et al., 2023), and their relative abundances increased with rising Fe^2+^ concentration (Figure 4B), suggesting a gradual increase in denitrification. In conclusion, SAD activity, anammox, and denitrification predominantly facilitated NO_3_^−^-N removal, while changes in the Fe^2+^ and Mn^2+^ ratios influenced the microbial community and distinct reactions involved.

Figure 4C illustrates the genus-level correlation analysis performed between the denitrification-related microbial communities and initial concentrations of Fe^2+^, Mn^2+^, NO_3_^−^-N removal rate (NRR), and SO_4_^2−^. 76.48% of the variation in microbial community structure at the genus level was represented by the horizontal axis, while 16% was denoted by the vertical axis. The horizontal axis was closely associated with the initial Fe^2+^ and Mn^2+^ concentrations, NRR, and SO_4_^2−^ production. In the system without Fe^2+^ and Mn^2+^ addition, the microbial community structure differed significantly from that of the systems containing 5 mM of various Fe^2+^ and Mn^2+^ ratios. The system was more sensitive to Fe^2+^ during NO_3_^−^-N removal, as evidenced by the substantial negative correlation between the NRR and SO_4_^2−^ production and the initial Fe^2+^ concentration and the positive correlation with the initial Mn^2+^ concentration. Introducing large quantities of Fe^2+^ appears to inhibit the growth of *Thiobacillus*, consequently affecting SAD function. The negative correlation with the initial Fe^2+^ concentration and the positive correlation with the initial Mn^2+^ concentration for the relative abundance of *Thiobacillus* evidences this relationship. The significant positive correlation between the NRR and relative abundance of *Thiobacillus* implies that SAD is primarily responsible for removing NO_3_^−^-N from the system, which is consistent with prior research findings (Pang and Wang, 2021).

### 3.5 Effects of Fe^2+^ and Mn^2+^ coexistence on the abundance of relevant functional genes in the SAD system

**Figure 5:**
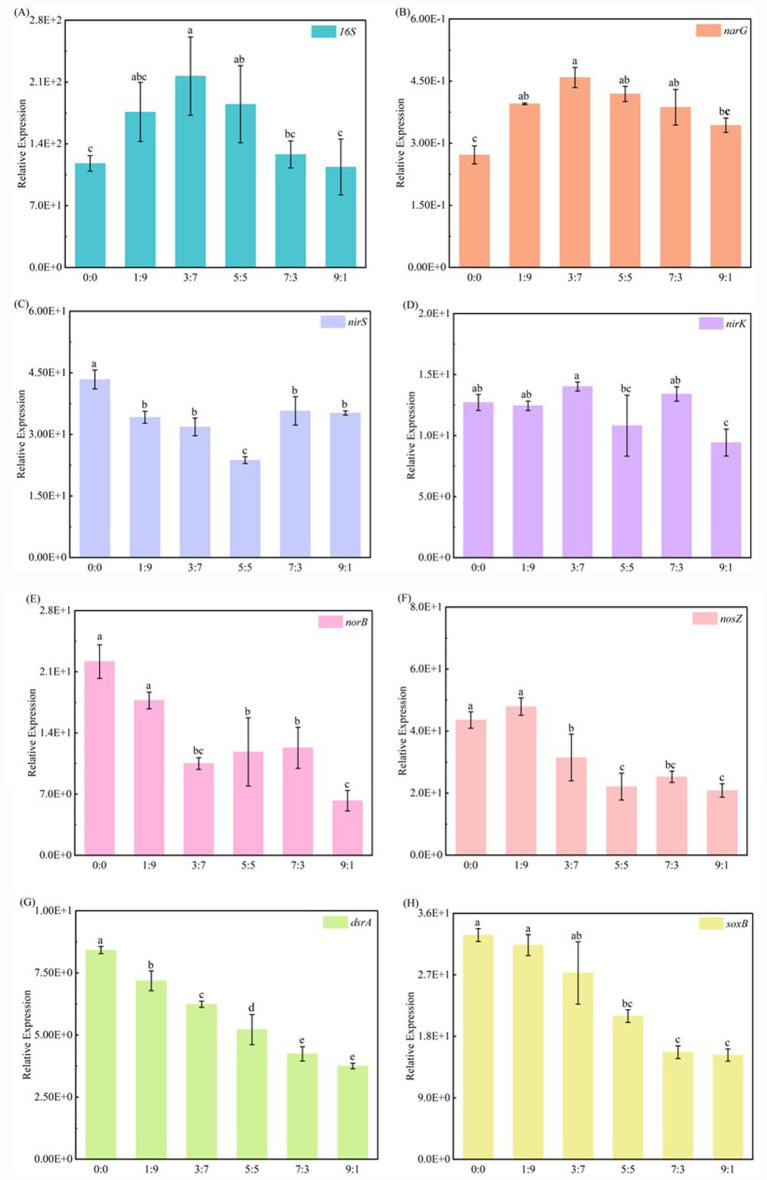
Relative expression of bacterial *16S rRNA* **(A)**, *narG* **(B)**, *nirS* **(C)**, *nirK* **(D)**, *norB* **(E)**, *nosZ* **(F)**, *dsrA* **(G)**, and *soxB* **(H)** genes in the system with coexisting Fe^2+^ and Mn^2+^.

Figure 5 illustrates the impact of coexisting Fe^2+^ and Mn^2+^ on the number of SAD-related functional genes. When the concentration of Fe^2+^ and Mn^2+^ increased and decreased, respectively, the relative expression of *16S rRNA* genes initially increased and subsequently declined (Figure 5A). This suggests that a moderate level of Fe^2+^ benefits microbial enrichment, whereas excessive Fe^2+^ concentrations inhibit microbial growth. The *narG* gene encoding nitrate reductase catalyzes the NO_3_^−^-N reducing to NO_2_^−^-N (Zhi and Ji, 2014). The relative expression of the *narG* gene in the systems with varying ratios of Fe^2+^ and Mn^2+^ was significantly higher than that in the system without Fe^2+^ and Mn^2+^ addition (Figure 5B). When the Fe^2+^: Mn^2+^ ratio was 3:7, the *narG* gene showed the highest relative expression. This suggests that the addition of varying ratios of Fe^2+^ and Mn^2+^ may enhance the ability of the SAD system to reduce NO_3_^−^-N to NO_2_^−^-N. Li et al. (2024) constructed a S-Mn carbonate ore denitrification (SMCD) reactor by combining Mn^2+^-rich manganese carbonate ore with SAD and discovered that the *16S rRNA* and *narG* gene abundances in the SMCD reactor were noticeably greater than those in the reactor without manganese carbonate ore addition.

Nitrite reductase catalyzes the NO_2_^−^-N reducing to NO, with the corresponding genes being *nirK* and *nirS*. Compared with the system without Fe^2+^ and Mn^2+^, the relative expression of the *nirS* gene substantially decreased with the addition of various Fe^2+^ and Mn^2+^ ratios (Figure 5C). Moreover, the relative expression of the *nirS* gene exhibited an initially decreasing and subsequently increasing trend in response to rising Fe^2+^ and falling Mn^2+^ concentrations. This suggests that increasing Fe^2+^ concentrations strongly inhibited *nirS* gene expression, whereas decreasing Mn^2+^ concentrations might have reinstated *nirS* gene expression. Among them, an Fe^2+^: Mn^2+^ ratio of 5:5 inhibited the relative expression of *nirS* by up to 45.32%. Compared to the system without Fe^2+^ and Mn^2+^ addition, the low proportion of Fe^2+^ in the system with coexisting Fe^2+^ and Mn^2+^ did not significantly impact the relative expression of the *nirK* gene. The relative expression of the *nirK* gene was only significantly suppressed when the Fe^2+^: Mn^2+^ ratio was 9:1, with a suppression rate of 25.84% (Figure 5D). Furthermore, the relative expression of the *nirK* gene in all systems was lower than that of the *nirS* gene, indicating that the *nirS* gene is the dominant gene responsible for reducing NO_2_^−^-N in the SAD system (Zhi and Ji, 2014).

In all systems (except the system with an Fe^2+^: Mn^2+^ ratio of 1:9), the relative expression of the *norB* gene, which encodes nitric oxide reductase and catalyzes the reduction of NO to N_2_O, was significantly decreased following the addition of varying Fe^2+^ and Mn^2+^ ratios (Figure 5E). This suggests that the coexisting Fe^2+^ and Mn^2+^ inhibit the reduction of NO to N_2_O in the SAD system. The Fe^2+^: Mn^2+^ ratio of 9:1 demonstrated the highest suppression of the relative expression of the *norB* gene, amounting to a 71.83% reduction. When the Fe^2+^: Mn^2+^ ratio was greater than 1:9 (Figure 5F), the relative expression of the *nosZ* gene was significantly lower than that in the system without Fe^2+^ and Mn^2+^ addition. This suggests that an increase in the Fe^2+^ concentration in the system with coexisting Fe^2+^ and Mn^2+^ inhibited the systemic reduction of N_2_O. The Fe^2+^: Mn^2+^ ratio of 9:1 exhibited the largest inhibition (52.23%) of the relative expression of the *nosZ* gene. Zheng et al. (2024) investigated the relationship between the autotrophic denitrification rate and Fe/N ratio. They found that increasing the ratio from 2 to 4 decreased the relative abundances of the *nosZ* and *norB* genes. In conclusion, adding the appropriate Fe^2+^ and Mn^2+^ addition can boost microbial diversity and abundance, enhance *narG* gene expression, and promote the NO_3_^−^-N reducing to NO_2_^−^-N. However, by suppressing the expression of the *nirS* and *nosZ* genes, adding 5 mM of various Fe^2+^ and Mn^2+^ ratios prevented the NO_2_^−^-N and N_2_O conversing, thereby resulting in the NO_2_^−^-N and N_2_O accumulation.

Consistent with the experimental results of Pang and Wang (2021), the relative expression of Sulfur-oxidizing genes (*dsrA* and *soxB*) gradually decreased as the concentration of Fe^2+^ increased (Figures 5G,H). The *dsrA* gene is engaged in the oxidation of S^0^ to SO_3_^2−^, with S^0^ and SO_3_^2−^ able to subsequently react to generate S_2_O_3_^2−^ (Bobadilla Fazzini et al., 2013). Additionally, SOB possessing the *soxB* gene can oxidize S_2_O_3_^2−^ to SO_4_^2−^ (Chen et al., 2020). Compared with the system without Fe^2+^ and Mn^2+^, the relative expression of the *dsrA* gene was considerably lower in systems with varying Fe^2+^ and Mn^2+^ ratios. However, the relative expression of the *soxB* gene was considerably reduced solely when the Fe^2+^ concentration exceeded 1.5 mM (Fe^2+^: Mn^2+^ = 3:7). Furthermore, the relative expression of the *soxB* gene corresponded to the production of SO_4_^2−^ in Section 3.2 (Figure 2A) and the relative abundance of the bacterial community in Section 3.4 (Figures 4A,B). These results indicate that a high Fe^2+^ concentration in the system with coexisting Fe^2+^ and Mn^2+^ inhibits the expression of the *soxB* gene, which lowers the relative abundance of the SOB genus *Thiobacillus*, ultimately resulting in decreased SO_4_^2−^ production.

### 3.6 Driver analysis of the SAD system with coexisting Fe^2+^ and Mn^2+^

**Figure 6:**
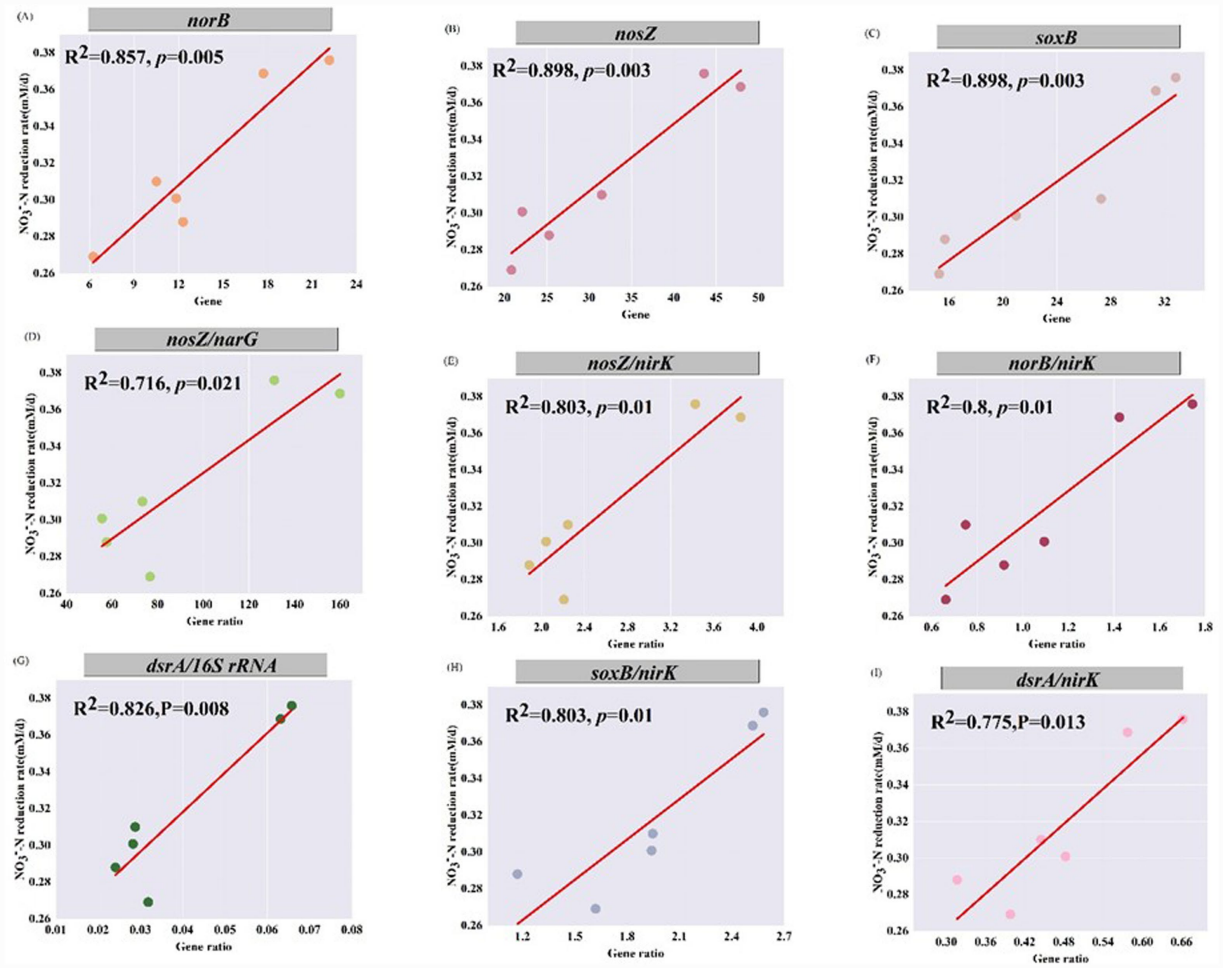
Relationship between the NO_3_^−^-N removal rate and relative expression of related genes **(A–C)** and their ratios **(D–I)** in the SAD systems with coexisting Fe^2+^ and Mn^2+^.

To identify the primary driving factors of the NO_3_^−^-N removal in the SAD system with 5 mM of varying ratios of coexisting Fe^2+^ and Mn^2+^, using a linear regression analysis to determine the relationship between the removal rate of NO_3_^−^-N in the SAD system and relative expression of relevant genes. Figure 6 presents the results, with the linear fitting line indicated by the straight red line. The figure illustrates a significant positive correlation between the removal rate of NO_3_^−^-N in the SAD system and relative expression of *norB*, *nosZ*, *soxB*, *nosZ*/*narG*, *nosZ*/*nirK*, *norB*/*nirK*, *dsrA*/*16S rRNA*, *soxB*/*nirK*, and *dsrA*/*nirK* (*p* = 0.003–0.021). The NO_3_^−^-N reducing was mediated by *narG*, the NO_2_^−^-N reducing by *nirK*, the NO reducing by *norB*, and the N_2_O reducing by *nosZ*. Consequently, *nosZ*/*narG*, *nosZ*/*nirK*, and *norB*/*nirK* denote the degree of complete denitrification for NO_3_^−^-N reduction to N_2_ (Pang and Wang, 2021), thereby suggesting that higher levels of complete denitrification are more favorable for the denitrification of the SAD system. Conversely, incomplete denitrification produces intermediates that are toxic to microorganisms, including NO_2_^−^-N. This lowers the N removal performance of the system, in which NO reduction and N_2_O reduction are the rate-limiting reactions.

Because *dsrA* is involved in S^0^ oxidation, *dsrA*/*16S rRNA* indirectly indicates the capacity of the system to oxidize S^0^ to SO_3_^2−^ and the relative abundance of SOB in the system. As previously mentioned, the addition of varying ratios of Fe^2+^ and Mn^2+^ reduced the relative abundance of the SOB *Thiobacillus* (Figure 4B) and the relative expression of Sulfur-oxidizing genes (*dsrA* and *soxB*) (Figures 5G,H). This resulted in the inhibition of the sulfur oxidation process within the system, potentially reducing the denitrification effect of the SAD system. *soxB* is crucial for S_2_O_3_^2−^ oxidation, and the rate of denitrogenation in the SAD system was positively correlated with its relative expression (Figure 6C). Therefore, the couple of NO_2_^−^-N reduction and sulfur oxidation (S^0^, S_2_O_3_^2−^) are reflected in *soxB*/*nirK* and *dsrA*/*nirK* (Pang and Wang, 2021). As described in Sections 3.1 and 3.2, SO_4_^2−^ production in the SAD system was positively correlated with the NO_3_^−^-N removal rate, implying that the denitrification process was coupled with the sulfur oxidation process in the system. In summary, denitrogenation in the SAD system was primarily driven by *nosZ*/*narG*, *nosZ*/*nirK*, *norB*/*nirK*, *dsrA*/*16S rRNA*, *soxB*/*nir*K, and *dsrA*/*nirK* in the coexisting Fe^2+^ and Mn^2+^, whereas the rate-limiting reactions were NO reduction and N_2_O reduction.

## 4 Conclusion

In the SAD system, the removal rate of NO_3_^−^-N on Day 6 gradually declined from 92.73% (absence of Fe^2+^ and Mn^2+^) to 60.96% (Fe^2+^: Mn^2+^ = 9:1) when 5 mM of varying ratios of Fe^2+^ and Mn^2+^ coexisted. As Fe^2+^ and Mn^2+^ concentrations increased and decreased, respectively, SO_4_^2−^ generation declined, Fe^2+^ removal gradually decreased, and Mn^2+^ removal gradually increased. Furthermore, when Fe^2+^ and Mn^2+^ coexisted, both NO_2_^−^-N and N_2_O accumulated in the SAD system. Fe^2+^ and Mn^2+^ coexistence inhibited the denitrification of SAD system by lowering the pH, relative abundance of *Thiobacillus*, and expression of denitrifying (*nirS*, *no*rB, and *nosZ*) and sulfur-oxidizing (*dsrA* and *soxB*) genes. *nosZ*/*narG*, *nosZ*/*nirK*, *norB*/*nirK*, *dsrA*/*16S rRN*A, *soxB*/*nirK*, and *soxB*/*nirK* ratios were the primary drivers of N removal from the SAD system with coexisting Fe^2+^ and Mn^2+^. NO reduction and N_2_O reduction were the rate-limiting processes. To gain deeper insights into the impact of Fe^2+^ and Mn^2+^ coexistence on SAD and to explore whether other autotrophic denitrification pathways coexist in the system, future experiments could measure the concentrations of iron and manganese products and monitor parameters such as redox potential.

## Data Availability

The data presented in the study are deposited in the Sequence Read Archive repository, accession number PRJNA1411803.
