# Supplementary material for: Coexistence of Fe2+ and Mn2+ inhibits nitrate removal in sulfur autotrophic denitrification systems
Source: Front Microbiol. 2026 Feb 25;17:1739270. doi: 10.3389/fmicb.2026.1739270 (PMC12978155; doi:10.3389/fmicb.2026.1739270)
Supplement: Supplementary file 1 [file Table_1.docx]

**Supplementary Material**

**Coexistence of Fe^2+^and Mn^2+^ inhibits** **nitrate removal in sulfur autotrophic denitrification systems**

Pengling Chen^a^, Xuejiao Huang ^a,b^*, Zhaojie Jiang^a^, Xiaofang Nong^a^, Chunmin Xie^b^

*^a^ Guangxi Key Laboratory of Agro-Environment and Agro-Products Safety, College of Agriculture, Guangxi University, Nanning, 530004, China*

*^b^ Guangxi Bossco Environmental Technology., Nanning, 530007, China*

** Corresponding author. E-mail address: hxuejiao0412@sina.com (X. Huang).*

Table S1 The forward and reverse primers of genes

| Genes | Primers | Primer sequence（5' to 3'） | Reference |
| --- | --- | --- | --- |
| *16S rRNA* | 338F  806R | ACTCCTACGGGAGGCAGCAG  GGACTACHVGGGTWTCTAAT | (Muyzer et al., 1993) |
| *narG* | 1960m2f  2050m2r | TAYGTSGGGCAGGARAAACTG  CGTAGAAGAAGCTGGTGCTGTT | (Lopez-Gutierrez et al., 2004) |
| *nirS* | cd3aF  R3cd | GTSAACGTSAAGGARACSGG  GASTTCGGRTGSGTCTTSAYGAA | (Kandele et al., 2006) |
| *nirK* | nirK876  nirK1040 | ATYGGCGGVAYGGCGA  GCCTCGATCAGRTTRTGGTT | (Henry et al., 2004) |
| *norB* | cnorB2F  cnorB6R | GACAAGNNNTACTGGTGGT  GAANCCCCANACNCCNGC | (Braker and Tiedje, 2003) |
| *nosZ* | nosZ2F  nosZ2R | CGCRACGGCAASAAGGTSMSSGT  CAKRTGCAKSGCRTGGCAGAA | (Henry et al., 2006) |
| *soxB* | 710F  1184R | ATCGGYCAGGCYTTYCCSTA  MAVGTGCCGTTGAARTTGC | (Tourna et al., 2014) |
| *dsrA* | dsr1-F  dsr-R | ACSCACTGGAAGCACGGCGG  GTGGMRCCGTGCAKRTTGG | (Leloup et al., 2007) |
| *rpoB* | rpoBF  rpoBR | GGGTGATCTTACGGTGCTGT  GCCATCTTCGGTGAGAAGAG | (Adékambi et al., 2009) |

The setup for the qPCR amplification program was as follows: (1) pre-denaturation stage, 95 °C for 30 s (1 cycle); (2) PCR amplification (45 cycles) comprising denaturation at 95 °C for 5 s, annealing at 59 °C for 30 s, and extension at 72 °C for 30 s; (3) Melting stage comprising heating to 95 °C for 5 s, cooling to 60 °C for 1 min, and reheating to 95 °C for 1 cycle (fluorescence acquisition); (4) Final cooling to 50 °C for 30 s (1 cycle).

**Reference**

Adékambi, T., Drancourt, M., Raoult, D.(2009). The *rpoB* gene as a tool for clinical microbiologists. *Trends in Microbiology*, 17(1), 37-45. <https://doi.org/10.1016/j.tim.2008.09.008>.

Braker, G., Tiedje, J.M.(2003). Nitric oxide reductase (*norB*) genes from pure cultures and environmental samples. *Applied and Environmental Microbiology*, 69(6), 3476-3483. <https://doi.org/10.1128/AEM.69.6.3476-3483.2003>.

Henry, S., Baudoin, E., López-Gutiérrez, J.C., Martin-Laurent, F., Brauman, A., Philippot, L.(2004). Quantification of denitrifying bacteria in soils by *nirK* gene targeted real-time PCR. *Journal of Microbiological Methods*, 59(3), 327-335. <https://doi.org/10.1016/j.mimet.2004.07.002>.

Henry, S., Bru, D., Stres, B., Hallet, S., Philippot, L.(2006). Quantitative detection of the *nosZ* gene, encoding nitrous oxide reductase, and comparison of the abundances of *16S rRNA*, *narG*, *nirK*, and *nosZ* genes in soils. *Applied and Environmental Microbiology*, 72(8), 5181-5189. <https://doi.org/10.1128/AEM.00231-06>.

Kandeler, E., Deiglmayr, K., Tscherko, D., Bru, D., Philippot, L.(2006). Abundance of *narG*, *nirS*, *nirK*, and *nosZ* genes of denitrifying bacteria during primary successions of a glacier foreland. *Applied and Environmental Microbiology*, 72(9), 5957-5962. <https://doi.org/10.1128/AEM.00439-06>.

Leloup, J., Loy, A., Knab, N.J., Borowski, C., Wagner, M., Jørgensen, B.B.(2007). Diversity and abundance of sulfate-reducing microorganisms in the sulfate and methane zones of a marine sediment, Black Sea. *Environmental Microbiology*, 9(1), 131-142. <https://doi.org/10.1111/j.1462-2920.2006.01122.x>.

Lopez-Gutierrez, J.C., Henry, S., Hallet, S., Martin-Laurent, F., Catroux, G., Philippot, L.(2004). Quantification of a novel group of nitrate-reducing bacteria in the environment by real-time PCR. *Journal of Microbiological Methods*, 57(3), 399-407. <https://doi.org/10.1016/j.mimet.2004.02.009>.

Muyzer, G., Dewaal, E.C., Uitterlinden, A.G.(1993). Progilling of complex microbial populations by denaturing gradient gel-electrophoresis analysis of polymerase chain reaction amplified genes coding for 16S ribosomal RNA. *Applied and Environmental Microbiology*, 59(3), 695-700. <https://doi.org/10.1128/aem.59.3.695-700.1993>.

Tourna, M., Maclean, P., Condron, L., O'Callaghan, M., Wakelin, S.A.(2014). Links between sulphur oxidation and sulphur-oxidising bacteria abundance and diversity in soil microcosms based on *soxB* functional gene analysis. FEMS Microbiology Ecology, 88(3), 538-549. <https://doi.org/10.1111/1574-6941.12323>.
